# Clinical improvement and reduction in serum calprotectin levels after an intensive exercise programme for patients with ankylosing spondylitis and non-radiographic axial spondyloarthritis

**DOI:** 10.1186/s13075-016-1180-1

**Published:** 2016-11-25

**Authors:** Andrea Levitova, Hana Hulejova, Maja Spiritovic, Karel Pavelka, Ladislav Senolt, Marketa Husakova

**Affiliations:** 1 Department of Rheumatology, First Faculty of Medicine, Charles University and Rheumatology Institute, Prague, Czech Republic; 2 Charles University, Faculty of Physical Education and Sport, Prague, Czech Republic

**Keywords:** Axial spondyloarthritis, Non-radiographic, Ankylosing spondylitis, Exercise programme, Disease activity, Calprotectin

## Abstract

**Background:**

The efficacy of exercise therapy for ankylosing spondylitis (AS) is well-documented, but dearth of information is for non-radiographic axial spondyloarthritis (nr-axSpA).

Biomarkers like serum calprotectin, interleukins IL-6, IL-17 and tumour necrosis factor (TNF)-α may reflect the disease activity of axial spondyloarthritis (axSpA). In this study, we investigated clinical and laboratory parameters of both axSpA subgroups in response to intensive physical exercise.

**Methods:**

Altogether, 46 patients with axSpA, characterised according to the Assessment of SpondyloArthritis International Society criteria as having nr-axSpA or AS underwent 6-month exercise programme. Clinical outcomes of disease activity, Bath AS Disease Activity Index (BASDAI), AS Disease Activity Index (ASDAS-CRP), mobility, Bath AS Metrology Index (BASMI) and function, Bath AS Functional Index (BASFI) were evaluated at baseline and at the end of the exercise programme. Serum IL-6 and IL-17, TNF-α and calprotectin were measured via ELISA. The clinical and laboratory data of 29 control axSpA patients were used for the evaluation of the results.

**Results:**

In all axSpA patients, the ASDAS-CRP (2.10 ± 0.12 to 1.84 ± 0.11, *p* <0.01) and BASMI (1.28 ± 0.14 to 0.66 ± 0.84, *p* <0.0001) improved after 6 months of exercise therapy. There was a significant improvement in the ASDAS-CRP in the nr-axSpA subgroup (2.01 ± 0.19 to 1.73 ± 0.16, *p* <0.05) and in the BASMI in both, the nr-axSpA and the AS subgroups (1.09 ± 0.12 to 0.47 ± 0.08, *p* <0.0001 and 1.43 ± 0.24 to 0.82 ± 0.23, *p* <0.0001, respectively). Both, ASDAS-CRP and BASDAI, were significantly improved in the exercise axSpA group compared to the control axSpA group (mean -0.26 vs. -0.13 and -0.49 vs. 0.12, respectively, all *p* <0.05). Only calprotectin was significantly reduced after the exercise programme in nr-axSpA and AS patients (from 2379.0 ± 243.20 to 1779.0 ± 138.30 μg/mL and from 2430.0 ± 269.70 to 1816.0 ± 148.20 μg/mL, respectively, all *p* <0.01). The change in calprotectin was more profound in the axSpA intervention group (mean -604.56) than in the control axSpA (mean -149.28, *p* <0.05).

**Conclusion:**

This study demonstrated similar efficacy for an intensive exercise programme in both nr-axSpA and AS patients. A significant decrease in serum calprotectin levels in both subgroups of axSpA patients after the exercise programme reflected an improvement in the disease activity and spinal mobility.

**Electronic supplementary material:**

The online version of this article (doi:10.1186/s13075-016-1180-1) contains supplementary material, which is available to authorized users.

## Background

Axial spondyloarthritis (axSpA) is thought to consist of two subtypes of one disease that share similar features, such as inflammatory back pain, peripheral arthritis, enthesitis, dactylitis, various extraarticular manifestations and a genetic background, which is evident in a common family history and HLA B27 positivity. The hallmarks of axSpA in particular are inflammation in the sacroiliac joints (SIJ) and in the tendons of the axial skeleton [1, 2].

The radiographic axSpA variant ankylosing spondylitis (AS) has been well-characterised by the modified New York criteria, which include evidence of typical bone changes on conventional radiographs of the SIJ [1]. The non-radiographic subgroup, nr-axSpA, on the other hand, can manifest as a spectrum of spondyloarthritic signs together with either human leukocyte antigen (HLA)-B27 positivity or inflammation of the SIJ, which can be detected by magnetic resonance imaging (MRI) but not by conventional radiography [2]. Although distinct differences, such as female-to-male ratio, impaired function, mobility and levels of C-reactive protein (CRP) distinguish AS from nr-axSpA, an analogous disease course and clinical activity are common to both subgroups, and nr-axSpA is considered to be an earlier stage of AS [3]. Similarly, an identical management approach to both subgroups of axSpA is proposed in the guidelines of the Assessment of SpondyloArthritis International Society (ASAS) [4].

Reduced disease activity and improved quality of life are the main treatment goals for patients with axSpA. Pharmacological agents, including non-steroidal anti-inflammatory drugs (NSAIDs) and tumour necrosis factor (TNF)-α inhibitors (TNFi), are efficacious in a majority of patients even though the effect of current treatment on structural progression remains unclear [5]. A combination of both pharmacological and non-pharmacological interventions such as physiotherapy and education, however, appears to be more effective in controlling the disease activity [6, 7] and periodic exercise ameliorates the effect of long-term AS on disability and deformity [8]. Current recommendations for AS exercise therapy focus not only on the modification of AS musculoskeletal sequelae but also on the secondary consequences of AS such as balance disturbances and cardiorespiratory abnormalities [7]. However, the efficacy of exercise therapy per se in patients with nr-axSpA has not yet been elucidated.

Disease activity and severity of axSpA are well-documented in a number of established indices, but only the AS Disease Activity Score (ASDAS) reflects inflammatory activity with entry values of CRP and patient-reported valuations [9]. Although increased CRP alone is not a specific condition of high AS disease activity and has only been found to be common in 40–50% of patients [10], they serve as a biomarker for the evaluation of inflammation in daily practice. Several other biomarkers, cytokines in particular, are purported to have a connection to disease activity and severity. Interleukins such as IL-6 and IL-17 and TNF-α are presumed to play an important role in axSpA pathogenesis due to the initiation and maintenance of inflammation [11–13]. In addition to the overproduction of IL-6 and TNF-α in SIJ tissues [11, 12], patients with greater disease activity were also found to have elevated circulating levels of IL-6, IL-17 and TNF-α [14, 15]. Similarly, calprotectin (S100A8/S100A9 heterodimer) is mainly produced by monocytes that infiltrate the inflamed synovium during spondyloarthritis [16], and elevated systemic levels reflect global autoimmune inflammation [17].

In this study, we carried out a 6-month exercise and education programme for patients who were suffering from both nr-axSpA and AS. We investigated the impact of intensive physical training on disease activity in the whole group and, in particular, on differences between the two defined subgroups. Variations in biomarker serum levels as affected by exercise were also evaluated.

## Methods

### Patients

#### AxSpA with exercise programme

A total of 46 patients with axSpA who were attending the Institute of Rheumatology in Prague were included in this study. All of the patients fulfilled the ASAS classification criteria for axSpA [2] and according to these criteria were characterised as having nr-axSpA (n = 23) or AS (n = 23). Patients in the nr-axSpA group had clinical signs and symptoms of SpA and were either positive for the HLA-B27 antigen and/or had inflammatory changes in the SIJ on MRI (73.9%) but no changes on radiography. All of the AS patients fulfilled the modified New York criteria for the diagnosis of AS, including the radiographic criterion [1]. Eight patients with AS with a longer disease duration (14.76 ± 2.17 and 9.50 ± 2.98 years since the first symptoms and diagnosis, respectively) had spinal involvement (25%), and fifteen patients with AS with shorter disease duration (11.93 ± 1.80 and 5.30 ± 1.26 years since the first symptoms and diagnosis, respectively) only had SIJ involvement. All of the patients with axSpA were fully or partially employed, mostly (95.7% of both nr-axSpA and AS) as “white-collar” workers [18]. The inclusion criteria for the study were stable disease and treatment for at least 6 months prior to baseline. During the entire study, transient changes in the dose of NSAIDs were allowed. Patients must not have received oral or intraarticular glucocorticoids during the study.

#### Control group - patients with axSpA with no intervention

The control group consisted of 29 age-matched and sex-matched patients with axSpA. Out of the control group, 15 subjects had nr-axSpA. All of the patients from the control group were selected retrospectively from the axSpA cohort in Prague, and the following parameters were required: dates of two medical examinations (marked as visits 1 and 2) that occurred concurrently with the ongoing study, stable disease activity and treatment for at least 6 weeks prior to visit 1, and no changes in pharmacological or other therapy between visits 1 and 2. In particular, subjects from the control group must not have undergone physical therapy, physical exercise or spa treatment for at least 6 weeks prior to baseline and between the two consecutive visits. Daily home exercise without any supervision and mobilisation techniques or massage was allowed. All of the control patients with axSpA were fully or partially employed, and the percentages of white-collar workers [18] were 93.4% for nr-axSpA and 85.7% for AS (not significantly different from groups with exercise).

### Intervention

This was a 6-month single-centre study that started in September 2013. All of the subjects from the intervention group underwent outpatient group physiotherapy for 60 minutes twice a week and a daily home-based exercise programme. The absence of more than two consecutive sessions of outpatient physiotherapy excluded patients from further participation in the study. The supervised exercise unit was made up of three sections: warm-up at the beginning, the main part and cool-down at the end. During the warm-up, cardiorespiratory fitness methods were used. These methods focused on concentration, core strengthening and spinal traction. The following exercises were employed: spinal (rotatory) exercises developed by Cumpelik [19], an educational back school method [20] and McKenzie therapy [21], posture correction according to the Brügger concept [22], elements derived from pilates [23] and muscle stretching. The major part was dedicated to the practice of correct activation of deep core stabilisation and the precise involvement of muscles in motion and balance training, particularly using BOSU balls, which are an established method for the encouragement of abdominal muscle activity [24] as a tool for stretching, lumbar strengthening and hip-joint mobility [25]. The final part (cool-down) consisted of techniques of relaxation, breathing exercises and elements of yoga. Additionally, physiotherapy education for homework - practical training in postural correction exercises and motivation to change the daily routine was used at the end of the exercise unit.

### Outcome measurements

#### Clinical

The clinical examination of signs of axSpA, including extraarticular manifestation, was carried out in all patients before and a few days (maximum 7 days) after the exercise programme termination. The measurement tools for disease activity and severity evaluation were the Bath AS Disease Activity Index (BASDAI) [26], Bath AS Functional Index (BASFI) [27], the 10 point Bath AS Metrology Index (BASMI) [28] and the AS Disease Activity Index (ASDAS)-CRP [9]. BASMI was rated by the same physiotherapist at the beginning and at the end of the exercise programme. In the control axSpA group, in which no intervention was performed, the disease activity was assessed using the BASDAI, BASFI and ASDAS-CRP in two consecutive visits after 6 months. The BASMI, however, was not available for this analysis.

#### Biomarkers

In the intervention group, serum sampling was carried out at baseline and at the end of the study on the same date as the clinical evaluation. Fasting blood samples were immediately centrifuged and stored at -80 °C and were removed before analysis, which was carried out on all intervention group samples simultaneously. In the axSpA control group, blood was withdrawn at each visit, and the samples were treated as above. CRP was measured with the immuno-turbidimetric test using a biochemical analyser (Olympus, model AU 400 Japan). IL-6, IL-17, TNF-α (RayBiotech, Inc., Norcross GA, USA) and calprotectin (Bühlmann Laboratories AG, Schőnenbuch, Switzerland) were analysed using a commercially available enzyme-linked immunosorbent assay (ELISA) according to the manufacturer`s protocols. All of the assays were run in duplicate. The inter-assay and intra-assay reliability of the IL-6 and IL-17 and TNF-α assays was <12% and <10%, respectively, and the inter-assay and intra-assay reliability of calprotectin was 5.8% and 4.3%, respectively. Absorbance was detected using the Sunrise ELISA reader (Tecan Group Ltd., Salzburg, Austria) with 450 nm as the primary wavelength. The sensitivity for IL-6, IL-17, TNF-α and calprotectin was determined to be 3 pg/mL, 80 pg/mL, 30 pg/mLl and 0.4 μg/mL, respectively.

### Statistical analysis

The data are expressed as the means ± standard deviations. Qualitative variables were tested using the chi-squared or Fisher’s exact test. Biomarker data were evaluated using the Friedman test. The variables at baseline and at the end of the exercise programme were analysed using the non-parametric Wilcoxon paired test. The differences between groups were analysed using the non-parametric unpaired test and the Mann-Whitney *U* test. Multiple comparisons were corrected using Monte Carlo exact significance testing. Ordinary least squares (OLS) regression models were used for predictive analysis. The Spearman correlation coefficient was used for correlation analysis. *P* values less than 0.05 were considered statistically significant. The SPSS (version 22) and GraphPad Prism 7 programmes were used for all analyses.

## Results

### Clinical characteristics of the cohort

A total of 40 patients with nr-axSpA (n = 18) or AS (n = 22) completed the exercise and educational programme. Six patients were excluded for the following reasons: current inflammatory bowel disease (IBD) in the nr-axSpA group, exacerbation of peripheral arthritis requiring a change in long-term therapy (AS group), and four patients who missed two or more consecutive sessions were also excluded (nr-axSpA group). At baseline, spondyloarthritic signs (HLA B27 positivity, extraarticular manifestation, hip involvement) and treatment were represented equally in both groups, and there was no significant difference in age, body mass index (BMI) or smoking history. Patients characterised as nr-axSpA, however, had significantly shorter disease duration since the occurrence of first symptoms and diagnosis (7.10 ± 2.19 versus 12.27 ± 1.42, *p* <0.001 and 1.81 ± 0.52 versus 5.84 ± 1.17, respectively, all *p* <0.05) and a trend of female predominance was observed (Table 1). During the exercise therapy, there were no significant differences in BMI, peripheral arthritis or the need to change long-term medication in either group. Similarly, the rate of exacerbation of the extraarticular manifestation was equal in the nr-axSpA and AS groups, and no patients developed clinical signs of colitis during the exercise therapy.

**Table 1:** Demographic and clinical characteristics of patients with axSpA participating in exercise therapy

	All (n = 40)	AS (n = 22)	nr-axSpA (n = 18)	*P* value
Age, years	36.78 ± 1.09	36.91 ± 1.16	36.61 ± 2.03	ns
Gender (% female)	32.5	18.2	50.0	0.05
BMI at baseline	25.25 ± 0.44	25.51 ± 0.76	25.02 ± 0.50	ns
Disease duration since first symptoms (years)	9.94 ± 1.31	12.27 ± 1.42	7.10 ± 2.19	˂0.001
Disease duration since diagnosis (years)	4.03 ± 0.75	5.84 ± 1.17	1.81 ± 0.52	˂0.01
HLA-B27 positivity (%)	90.0	90.9	88.8	ns
History of uveitis/exacerbation (%)^a^	35.0/7.5	27.7/9.1	44.4/5.5	ns
History of peripheral arthritis/exacerbation (%)^a^	42.5/2.5	45.5/4.5	38.9/0	ns
Hip involvement (%)	17.5%	22.7%	11.1%	ns
Therapy - NSA (daily use/on demand) (%)	100 (27.5/72.5)	100 (18.2/81.8)	100 (38.9/61.1)	ns
NSA changes during exercise therapy/changes to daily use (%)	7.5/2.5	0/0	16.7/5.5	ns
Therapy - sulfasalazin (%)	10.0	4.5	16.6	ns
Therapy – biological agents (%)^b^	5.0	9.0	0	ns
Smokers ever/current	25.0/20.0	31.8/27.3	22.2/16.7	ns

^a^Exacerbations of uveitis/arthritis occurred during exercise intervention. No extraarticular signs other than uveitis were manifested. ^b^Only tumour necrosis factor alpha inhibitors (TNFi) were used in long-term therapy for high disease activity. Statistical analysis: *p* value was calculated for comparison between the ankylosing spondylitis (AS) and the axial non-radiographic spondyloarthritis (nr-axSpA) group using either the Mann-Whitney test or chi-squared/Fisher´s exact test. Data are characterised as mean ± standard deviation unless stated otherwise. *axSpA* axial spondyloarthritis, *BMI* body mass index, *NSA* non-steroidal antirheumatic drugs, *ns* not significant

Patients in the control axSpA group used sulfasalazine more often than those in the intervention axSpA group (44.8% vs. 10.0%, *p* < 0.05), and more patients with AS in the intervention group had more frequent history of peripheral arthritis, but the exacerbation of peripheral arthritis was not different from the control group during the study (45.5% and 4.5% vs. 7.2% and 0%, *p* < 0.05 and not significant (ns), respectively). None of the patients from the control group had any clinical signs of colitis at baseline or after 6 months (data not shown). There were no significant differences in any other clinical parameters between the two groups (Additional file 1).

### The intensive exercise intervention improved disease activity

In all of the patients with axSpA who were undergoing exercise therapy, the ASDAS-CRP score significantly decreased after 6 months of exercise (from 2.10 ± 0.12 to 1.84 ± 0.11, *p <* 0.01). Similarly, the BASMI values were also significantly lower at the end of the training programme compared to baseline (from 1.28 ± 0.14 to 0.66 ± 0.84, *p* < 0.0001) (Table 2). Additionally, there was a trend of a decline in the BASDAI (*p* = 0.06), but not in the BASFI, after 6 months of training (Table 2). The ASDAS-CRP score consists of four self-assessment questions, while the BASDAI score only reflects the patients’ outcome reports. In further analyses, we focused on changes in patients’ reports. For the ASDAS-CRP score, the following assessments decreased significantly: back pain (from 4.10 ± 0.34 to 2.97 ± 0.42, *p* < 0.001) and patients global (from 3.70 ± 0.36 to 2.50 ± 0.42, *p* < 0.05). Only the responses to the first two BASDAI questions improved in patients with axSpA after the exercise intervention (from 37.90 ± 3.50 and 35.70 ± 4.21 to 28.10 ± 3.35 and 25.80 ± 3.15, respectively, *p* < 0.05 for both comparisons).

**Table 2:** Clinical parameters and biomarker levels of axSpA at baseline and after 6 months of exercise therapy

	All axSpA			nr-axSpA			AS		
	(n = 40)			(n = 18)			(n = 22)		
	Baseline	6 months	*P* value	Baseline	6 months	*P* value	Baseline	6 months	*P* value
ASDAS-CRP	2.10 ± 0.12	1.84 ± 0.11	<0.01	2.01 ± 0.19	1.73 ± 0.16	<0.05	2.17 ± 0.16	1.93 ± 0.15	ns
BASDAI	2.78 ± 0.31	2.30 ± 0.25	ns	2.98 ± 0.28	2.27 ± 0.41	ns	2.63 ± 0.35	2.31 ± 0.32	ns
BASFI	1.06 ± 0.16	1.01 ± 0.14	ns	1.23 ± 0.28	1.73 ± 0.16	ns	0.92 ± 0.17	0.93 ± 0.18	ns
BASMI	1.28 ± 0.14	0.66 ± 0.84	<0.0001	1.09 ± 0.12	0.47 ± 0.08	<0.0001	1.43 ± 0.24	0.82 ± 0.23	<0.0001
CRP (mg/l)	4.67 ± 0.89	3.86 ± 0.82	ns	2.10 ± 0.48	2.16 ± 0.56	ns	6.76 ± 1.45	5.24 ± 1.37	ns
IL-6 (pg/ml)	10.31 ± 3.93	10.07 ± 4.60	ns	14.78 ± 8.35	15.40 ± 9.79	ns	6.48 ± 1.46	5.51 ± 1.63	ns
IL-17 (pg/ml)	323.50 ± 57.06	401.50 ± 75.87	ns	349.20 ± 82.55	285.30 ± 72.73	ns	301.80 ± 80.57	499.30 ± 123.10	ns
TNF-α (pg/ml)	65.22 ± 12.30	56.89 ± 10.61	ns	55.87 ± 10.49	45.03 ± 6.76	ns	72.49 ± 20.45	62.12 ± 18.06	ns
Calprotectin (ng/ml)	2408.0 ± 183.30	1800.0 ± 101.90	<0.001	2379.0 ± 243.20	1779.0 ± 138.30	<0.01	2430.0 ± 269.70	1816.0 ± 148.20	<0.01

The statistical significance was determined as a *p* value < 0.05; the paired non parametric test (Wilcoxon) was used for analysis of baseline versus 6 month data for each group. All data are characterised as mean ± standard deviation. *axSpA* axial spondyloarthritis, *nr-axSpA* non-radiographic axial spondyloarthritis, *AS* ankylosing spondylitis, *ASDAS-CRP* AS disease activity score, *CRP* C-reactive protein, *BASDAI* Bath AS disease activity index, *BASFI* Bath AS functional index, *BASMI* Bath AS metrology index, *IL* interleukin, *TNF* tumour necrosis factor, *ns* not significant

The measures of disease activity and severity (ASDAS-CRP, BASDAI, BASFI and BASMI) did not differ between groups at baseline or after 6 months of intervention. When all of the patients from the intervention cohort were divided into non radiographic and radiographic subgroups, the ASDAS-CRP decreased significantly in the nr-axSpA subgroup (from 2.01 ± 0.19 to 1.73 ± 0.16, *p* < 0.05) and non-significantly in the AS subgroup (from 2.17 ± 0.16 to 1.93 ± 0.15, p = 0.06). The BASMI score decreased after the intervention in both the nr-axSpA and AS subgroups (from 1.09 ± 0.12 to 0.47 ± 0.08 and from 1.43 ± 0.24 to 0.82 ± 0.23, respectively, *p* < 0.0001 for both comparisons) (Table 2). There were no significant differences between the two subgroups in the changes in ASDAS-CRP, BASDAI or BASMI over the exercise training (data not shown).

In the whole control group of patients with axSpA and both the nr-axSpA and AS subgroups without physical intervention, there were no significant changes in the ASDAS-CRP, BASDAI or BASFI scores between the two 6-month visits. The criterion “no change of therapy” was required to be included in this group, and this group served as a control group with stable disease. When we compared all three scores at visit 2 to the values of the BASDAI, BASFI and ASDAS-CRP of axSpA after physical intervention, no significant differences were found (see Additional file 1). However, when we looked for the ∆ASDAS-CRP and ∆BASDAI, we found a significant decrease in both parameters in the axSpA exercise group (-0.26 and -0.49, respectively) compared to axSpA with no intervention (-0.13 and 0.12, respectively), both *p* < 0.05 (Table 3).

**Table 3:** Comparison of the changes in disease activity scores and biomarkers levels within 6 months in the axSpA groups with and without exercise intervention

	All axSpA			nr-axSpA			AS		
	Exercise group (n = 40)	Without exercise (n = 29)	*P* value	Exercise group (n = 18)	Without exercise (n = 15)	*P* value	Exercise group (n = 22)	Without exercise (n = 14)	*P* value
∆ ASDAS-CRP	*-0.26* (0.58)	-0.13 (0.77)	<0.05	*-0.29* (0.44)	*0.03* (0.84)	ns	*-0.24* (0.68)	*-0.13* (0.72)	ns
∆ BASDAI	*-0.49* (1.40)	*0.12* (1.40)	<0.05	*-0.70* (1.48)	*0.42* (1.22)	ns	*-0.32* (1.34)	*0.12* (1.22)	ns
∆ CRP	*-0.81* (4.67)	*-0.33* (9.70)	ns	*0.06* (1.94)	*1.94* (12.20)	ns	*-1.52* (6.02)	*-2.76* (5.49)	ns
∆ Calprotectin	*-604.56* (1042.80)	*-149.28* (1255.70)	<0.05	*-599.42* (824.00)	*-190.33* (1180.60)	ns	*-608.68* (1211.20)	*-149.28* (1255.70)	ns

Statistical analysis: the comparison of the mean of changes in all parameters was performed using the non-parametric Mann-Whitney *U* test. The multiple comparisons were corrected using Monte Carlo exact significance testing. All data are characterised as mean and (standard deviation). *axSpA* axial spondyloarthritis, *nr-axSpA* non-radiographic axial spondyloarthritis, *AS* ankylosing spondylitis, *ASDAS-CRP* AS disease activity score, *CRP* C-reactive protein, *BASDAI* Bath AS disease activity index, ∆ changes in the scores/biomarkers values, *ns* not significant

### Calprotectin levels significantly decreased after exercise therapy in patients with axSpA

Overall, in the axSpA group, there was a significant difference (*p* < 0.001, Friedman test) in all biomarkers (CRP, IL-6, IL-17, TNF-α and calprotectin) at baseline and after 6 months of exercise therapy. However, of the biomarkers, only calprotectin significantly decreased in patients with axSpA during the study (from 2408.00 ± 183.30 to 1800.00 ± 101.90 μg/mL, *p* < 0.001) (Table 2).

There was a significant difference in CRP levels between the nr-axSpA and AS subgroups at baseline (2.10 ± 0.48 and 6.76 ± 1.45 mg/l, *p* < 0.001) and after the intervention (2.16 ± 0.56 and 5.24 ± 1.37 mg/l, *p* < 0.05), but the other biomarkers were comparable between both subgroups. On the other hand, CRP remained unchanged throughout the study, and only calprotectin significantly decreased after exercise therapy in both patients with nr-axSpA (from 2379.0 ± 243.20 to 1779.0 ± 138.3 μg/mL, p < 0.01) and in pPatients with AS (from 2430.0 ± 269.7 to 1816.0 ± 148.2 μg/mL, *p* < 0.01) (Table 2).

To exclude variation in calprotectin levels over time, an analysis of calprotectin levels in the control axSpA group with no intervention was performed.

Baseline calprotectin levels in the control axSpA (2749.0 ± 225.8), nr-axSpA (2514.0 ± 303.1) and AS (3001.0 ± 334.8) groups were similar to the intervention group and remained unchanged after 6 months in all axSpA (2579.0 ± 267.9), nr-axSpA (2324.0 ± 381.8) and AS (2852.0 ± 375.2) groups (data not shown). When the calprotectin levels at visit 2 in the control axSpA group were compared to the calprotectin levels in the axSpA group after the intervention (see previous), the calprotectin levels were lower in all intervention groups (axSpA, nr-axSpA and AS, *p* < 0.05, ns and *p* < 0.05, respectively). The changes in calprotectin levels were significantly and consistently lower in the axSpA exercise group than the axSpA control (mean -604.56 vs. mean -149.28, *p* < 0.05), see Table 3.

Next, we studied whether improvements in disease activity and severity measures (∆ASDAS-CRP, ∆BASDAI, ∆BASFI and ∆CRP) were associated with changes in calprotectin levels over time. Using linear regression analysis, we found a positive association between changes in CRP and calprotectin over 6 months. Change in CRP of one unit resulted in an increase in calprotectin of 68 units (regression coefficient 67.99, *p* < 0.01) (Additional file 2). This effect was found in all groups independent of exercise intervention or subgroup characterisation. Moreover, in all patients with axSpA irrespective of exercise therapy, changes in calprotectin positively correlated with the changes in CRP (*ρ* = 0.360, *p* < 0.01) and ASDAS-CRP improvement (*ρ* = 0.285, *p* < 0.05) (data not shown).

### The other biomarkers modestly reflected changes in scores for disease activity and mobility after exercise therapy

In all axSpA groups and in both the nr-axSpA and AS subgroups, we found positive correlation between ∆ASDAS-CRP and ∆CRP (Table 4). Of the other cytokines, only ∆TNF-α positively reflected improvement in the clinical variables of ASDAS-CRP and BASMI, particularly in the nr-axSpA group (Table 4). This finding was supported by the linear regression model, in which only the effect of ∆TNF-α on the ∆ASDAS-CRP and ∆BASMI was demonstrated in the nr-axSpA (regression coefficient 0.010 and 0.004, respectively, all *p* < 0.05, data not shown). On the other hand, there were no relationships between the selected biomarkers and ∆BASDAI (data not shown).

**Table 4:** Correlations between the clinical variables and laboratory markers in axSpA with exercise therapy

		∆ BASMI	∆ ASDAS-CRP	CRP baseline	IL-6 baseline	IL-17 baseline	TNF-α baseline	∆ CRP	∆ IL-6	∆ IL-17	∆ TNF-α
∆ BASMI	*ρ*	1	0.271	-0.017	0.236	-0.027	-0.311	0.003	-0.058	-0.049	0.417
All (n = 40)	*ρ*	.	ns	ns	ns	ns	0.09	ns	ns	ns	*p* < 0.05
∆ ASDAS-CRP	*ρ*	0.271	1	-0.231	0.391	-0.131	-0.02	0.406	-0.205	0.035	0.377
All (n = 40)	*ρ*	ns	.	ns	p < 0.05	ns	ns	*p* < 0.001	ns	ns	*p* < 0.05
∆ BASMI	*ρ*	1	0.774	-0.596	0.441	-0.230	-0.477	-0.098	-0.213	0.010	0.563
nr-axSpA (n = 18)	*ρ*	.	*p* < 0.001	*p* < 0.01	ns	ns	ns	ns	ns	ns	*p* < 0.05
∆ ASDAS-CRP	*ρ*	0.774	1	-0.187	0.388	0.028	-0.351	0.437	-0.249	-0.074	0.636
nr-axSpA (n = 18)	*ρ*	p < 0.001	.	ns	ns	ns	ns	*p* < 0.05	ns	ns	*p* < 0.05
∆ BASMI	*ρ*	1	0.007	0.233	0.099	0.217	-0.143	0.123	0.158	-0.110	0.273
AS (n = 22)	*ρ*	.	ns	ns	ns	ns	ns	ns	ns	ns	ns
∆ ASDAS-CRP	*ρ*	0.007	1	-0.328	0.356	-0.266	0.102	0.395	-0.147	0.154	0.186
AS (n = 22)	*ρ*	ns	.	ns	ns	ns	ns	*p* < 0.05	ns	ns	ns

Statistical analysis: correlation analyses were performed by Spearman correlation (*ρ*). Statistical significance was determined as *p* < 0.05. *axSpA* axial spondyloarthritis, *nr-axSpA* non-radiographic axial spondyloarthritis, *AS* ankylosing spondylitis, *ASDAS-CRP* AS disease activity score, *BASMI* Bath AS metrology index, *CRP* C-reactive protein, *IL* interleukin, *TNF* tumour necrosis factor, ∆ changes of the scores/biomarkers values, *ns* not significant

## Discussion

This study demonstrates for the first time the clinical effectiveness of intensive exercise therapy for both radiographic and non-radiographic forms of axSpA. Although the outcomes may be affected by several limitations, we suggest that there was a positive effect on mobility and clinical activity. In addition, it appears that the reduction in serum calprotectin might be related to the improved clinical outcome in patients with axSpA.

The recent recommendations of rehabilitation for patients with axSpA suggest that complex physical activities may be used to maintain good clinical status [7]. The general endocrine, immune and metabolic changes that occur during physical activities might, however, be stressful, and the effects of exercise on patients with early stages of axSpA have not yet been examined. In this study, during the exercise intervention, disease activity, as measured by the ASDAS-CRP and BASDAI, significantly improved over time in subgroups of patients with both radiographic and non-radiographic axSpA, which is in agreement with previous studies that have demonstrated improved disease activity after exercise in patients with AS [6, 23, 29–31]. Moreover, there was no difference in extraarticular or articular exacerbations in either axSpA subgroup, and our clinical data may support the efficacy and safety of physical therapy not only in patients with AS but also in patients with nr-axSpA.

The first limitation of this study is the relatively small number of patients in each subgroup and the inclusion of patients with stable disease only. However, the small number reflects the real situation that is present in daily clinical practice and the need to adjust the exercise programme for patients with unstable disease as needed. A second limitation might be the occupational history - most of the patients with axSpA who participated in this study were working in white-collar professions. Recently, Ramiro et al. demonstrated greater radiographic progression in “blue-collar” workers within 2 years, and this occupational feature could amplify the effect of ASDAS-CRP on disease progression [32]. In light of this finding, the evaluation of the safety and efficacy of physical therapy in blue-collar workers with axSpA or those who perform additional physical activities such as sport, requires larger future studies. The next limitation may be the absence of imaging of the inflammatory signs in the SIJ prior to and after exercise therapy. Recent data from the DESIR cohort suggest a relationship between inflammation of the SIJ as detected on MRI and the ASDAS score in male patients [33]. Thus, it can be suggested that improvement in clinical disease activity after exercise therapy can be associated with the amelioration of inflammatory lesions in the SIJ in patients with axSpA. Further studies should be performed to elucidate this hypothesis.

After physical therapy, mobility was improved in both in patients with nr-axSpA and those with AS. However, our axSpA control group was selected retrospectively, and the BASMI data were excluded from the analysis; thus, the change in BASMI cannot be compared. In patients with AS, the efficacy of supervised and home-based exercise on mobility is well-demonstrated [29–31], and a positive effect on BASMI after 24 weeks of an exercise programme has been identified in TNFi-naive patients with AS [34] and in those on TNFi treatment [6, 35]. Our study demonstrated for the first time the same benefit of exercise therapy on spinal mobility in patients with nr-axSpA and patients with AS. Although mobility scores in patients with AS are generally worse than in patients with nr-axSpA [10, 36, 37], given that 10–12% of patients with nr-axSpA progress to develop the form of the disease that is radiographically evident [10, 38], it seems appropriate to maintain mobility in the best form possible. In our group, the BASMI scores were similar in both groups at baseline and after exercise therapy - this distinction could be confirmed by a lower BASMI value in patients with AS (1.46 at baseline) compared to registries (2.84 and 2.0) [10, 36] and was probably due to the characterisation of patients with AS who were motivated by the training programme. However, there was no follow up after the end of the exercise programme, and thus, we were unable to demonstrate any long-term positive effects on the BASMI in the nr-axSpA or AS cohorts. Therefore, studies with longer follow-up periods are required in further research.

Increased serum calprotectin has been demonstrated in patients with other immune-mediated diseases, such as rheumatoid arthritis (RA) or psoriatic arthritis [39, 40], and in patients with AS [17, 41, 42] and nr-axSpA [41]. Serum calprotectin in patients with AS appears not to be influenced by medications such as NSAIDs or sulphasalazine [41, 43] and tends to be elevated in patients with AS with active peripheral arthritis [42]. In our study, baseline serum calprotectin was similar in patients with AS who were or were not on the exercise programme, although patients with AS who were not receivingexercise therapy had a more frequent history of peripheral arthritis.

To the best of our knowledge, we are the first to show a significant decrease in serum calprotectin after intensive physiotherapy in both nr-axSpA and patients with AS. As we have not studied the effect of this exercise programme on serum calprotectin in healthy individuals, we cannot be sure if this is a disease-specific effect or if the same might also occur in healthy subjects. Although skeletal muscles express calprotectin mRNA after contractions [44] and calprotectin plasma levels increase immediately and one hour after aerobic exercise in healthy individuals [45], there is no long-term effect of muscle contraction on systemic calprotectin. Kanda et al. performed a study in nine healthy volunteers who performed one-leg calf-raise exercises that included repetitive muscle contractions and found no significant differences in plasma or urine levels of IL-6, TNF-α and calprotectin immediately after and 96 hours after the exercise [46]. Similarly, Acar et al. recently found only a slight decrease in calprotectin levels in healthy subjects 8 weeks after they completed an exercise programme [47]. In agreement with our study, in patients with RA, the calprotectin levels decreased significantly and reflected improved disease activity and a decline in CRP [47].

In our study, the decrease in serum calprotectin levels was evident in both axSpA subgroups compared to control subjects with axSpA who underwent no exercise therapy. Interestingly, the synergistic effect of the decline in serum calprotectin and clinical improvement was found in two pharmacological studies in patients with AS, using TNFi (infliximab) [48] and anti-IL-17 (secukinumab) [49] treatments. However, in our study, regression analysis showed only a slight influence of exercise on serum calprotectin in patients with axSpA. This finding might be due to the small sample size. The regression analysis, on the other hand, supports the relationships between serum CRP and calprotectin, and those between ASDAS-CRP improvement and changes in calprotectin. Out of many biomarkers, serum calprotectin might represent a valuable tool for the assessment of disease activity improvement in patients with axSpA. In agreement with Turina et al. [48], our data also suggest that serum calprotectin may be a useful biomarker that reflects clinical improvement even in CRP-negative patients.

Calprotectin likely plays a role in axSpA pathogenesis and in the disease course. De Rycke et al. demonstrated 10-fold higher calprotectin in inflamed synovium than in serum [16]. Furthermore, the number of synovial monocytes and granulocytes that release calprotectin rapidly decreases after TNFi treatment in patients with peripheral SpA [50]. Recent data from the GESPIC cohort indicate that elevated serum calprotectin is an independent predictor of disease progression and syndesmophyte formation [51]. The precise immune mechanisms that occur in the inflamed joints during exercise are still unclear, but pharmacological studies suggest that decreased calprotectin serum levels might reflect local inflammation and possibly new bone formation [48–50]. However, larger studies based on registries and long-term observations are needed to evaluate the effect of physiotherapy on calprotectin and axSpA outcomes.

A possible limitation of our study is the absence of faecal calprotectin analysis. Faecal calprotectin has been shown to be elevated in patients with AS and SpA [41, 52], especially in patients with intestinal inflammatory changes [41] and in NSAID users [52]. Only 10% of patients with SpA develop IBD during the disease course [53], but 25–69% of patients without clinical IBD symptoms may have microscopic intestinal inflammation [43]. In our study, one patient was excluded due to a diagnosis of IBD, but none of the other participants underwent ileocolonoscopy because of a lack of clinical symptoms. Although the increased CRP and serum calprotectin appear to be predictive markers for intestinal involvement in SpA [41] and faecal calprotectin is a well-known marker of IBD activity [54], there is no correlation between serum and faecal calprotectin [41, 55]. A beneficial effect of mild exercise on the course of IBD has been suggested [56], but the relationship between intestinal inflammation and axSpA disease activity and progression in respect to physical activity requires further research.

Finally, in the evaluation of biomarkers after physical activity, we should bear in mind that exercise is a complex process that may lead to the release of mediators that influence metabolic, immune and neuroendocrine pathways. In relation to strenuous exercise, cytokines such as IL-6, IL-1 receptor antagonist, TNF-α and others are released over time [57] and regulate hormone, adaptive and innate immune cell activity. In the present study, interestingly, the changes in TNF-α correlated positively with the improvement in ASDAS-CRP and with the improvement in the mobility score but only in the nr-axSpA subgroup. There were no significant differences in calprotectin serum levels between patients with AS and those with nr-axSpA. We suggest that patients with axSpA who are in the early phase of the disease might respond to exercise differently from those with long-term disease duration and with different states of inflammation. It is well-known that both patients with nr-axSpA and those with AS respond positively to TNFi treatment [58]. Although the serum levels of TNF-α may not clearly reflect AS disease activity [59], it can be speculated that a decrease in TNF-α levels might serve as a biomarker of milder disease activity. In our study the levels of IL-17 increased, albeit not significantly, in the AS group. In healthy male subjects, physical activity such as high and mild-to-moderate training have not been shown to influence serum IL-17a [60], but another study in duathlon athletes suggests a potential role of the IL-17 axis in muscle damage after prolonged training [61]. On the other hand, women with multiple sclerosis were found to have lower levels of systemic IL-17 and a lower inflammatory status after an 8-week exercise programme [62]. The pivotal role of the IL-17 axis in axSpA pathogenesis has been suggested, and inhibition of IL-17 has recently been demonstrated to be safe and effective for AS treatment [49, 63]. In light of this, the variation in serum IL-17 after exercise therapy in the nr-axSpA and the AS group requires confirmation in further studies.

## Conclusion

In conclusion, this is the first study to show that exercise therapy is beneficial for both radiographic and non-radiographic subgroups of axSpA. In addition to better mobility, disease activity improved after an intensive exercise programme. This suggests that exercise should be an essential aspect of the axSpA treatment strategy, particularly in the early phases of the disease. In addition, a decrease in serum calprotectin levels reflected improvement in disease activity in both axSpA subgroups. Larger studies are needed to confirm our results.

## Additional files

Additional file 1: Demographic and clinical characteristics of patients with axSpA not on exercise therapy compared to patients with axSpA participating in the exercise intervention. Clinical and demographic data of control patients with axSpA are presented. Comparisons between control nr-axSpA and patients with AS and comparisons between patients with axSpA undergoing exercise therapy to control patients with axSpA with no intervention are included. (PDF 139 kb)
Additional file 2: The dependence of the change in calprotectin levels on the changes in clinical and laboratory parameters. This table presents the regression model of the dependent variable ∆calprotectin and the independent variables of clinical and laboratory parameters. Only ∆CRP significantly influenced ∆calprotectin. (PDF 198 kb)

## Abbreviations

AS: ankylosing spondylitis
ASAS: Assessment of SpondyloArthritis International Society
ASDAS: ankylosing spondylitis disease activity score
axSpA: axial spondyloarthritis
BASDAI: Bath Ankylosing Spondylitis Disease Activity Index
BASFI: Bath Ankylosing Spondylitis Functional Index
BASMI: Bath Ankylosing Spondylitis Metrology Index
BMI: body mass index
CRP: C-reactive protein
ELISA: enzyme-linked immunosorbent assay
IBD: inflammatory bowel disease
IL: interleukin
MRI: magnetic resonance imaging
nr-axSpA: non-radiographic axial spondyloarthritis
ns: not significant
NSAIDs: non-steroidal anti-inflammatory drugs
SIJ: sacroiliac joints
TNFi: tumour necrosis factor (TNF)-α inhibitors
TNF-α: tumour necrosis factor-α
