# Supplementary material for: Clinical improvement and reduction in serum calprotectin levels after an intensive exercise programme for patients with ankylosing spondylitis and non-radiographic axial spondyloarthritis
Source: Arthritis Res Ther. 2016 Nov 25;18:275. doi: 10.1186/s13075-016-1180-1 (PMC5124318; doi:10.1186/s13075-016-1180-1)
Supplement: Additional file 1: — Demographic and clinical characteristics of patients with axSpA not on exercise therapy compared to patients with axSpA participating in the exercise intervention. Clinical and demographic data of control patients with axSpA are presented. Comparisons between control nr-axSpA and patients with AS and comparisons between patients with axSpA undergoing exercise therapy to control patients with axSpA with no intervention are included. (PDF 139 kb) [file 13075_2016_1180_MOESM1_ESM.pdf]

Additional File 1

Demographic and Clinical Characteristics of axSpA patients without exercise therapy and comparison to axSpA patients with exercise intervention

	All (n=29)	AS (n=14)	nr-axSpA (n=15)	Comparison between AS and nr-xSpA	Comparison between Exercise group and Group without intervention*
Age	34.83±1.68	36.36±2.82	33.40±1.93	ns	ns[1], ns[2], ns[3]
Gender (% of Female)	31.0	21.4	40.0	ns	ns[1], ns[2], ns[3]
BMI visit 1	25.25±0.87	25.01±1.06	25.47±1.41	ns	ns[1], ns[2], ns[3]
Disease duration since First symptoms (years)	10.26±1.88	12.69±3.36	7.99±1.76	ns	ns[1], ns[2], ns[3]
Disease duration since Diagnosis (years)	3.31±0.80	3.93±1.20	2.73±1.09	ns	ns[1], ns[2], ns[3]
HLA B27 positivity (%)	96.5	100	93.3	ns	ns[1], ns[2], ns[3]
History of uveitis/exacerbation (%) **	37.9 / 10.34	35.7 / 14.3	40.0 / 6.7	ns	all ns[1], ns[2], ns[3]
History of peripheral arthritis/exacerbation (%) **	24.1 / 0	7.2 / 0	40.0 / 0	ns	ns[1]/ns[1], p[2]=0.014/ns[2], ns[3]/ns[3]
Hip involvement (%)	24.1	21.4	26.7	ns	ns[1], ns[2], ns[3]
Therapy - NSA (daily use/on demand) (%)	96.5 (17.8/82.2)	93 (15.4/84.6)	100 (20/80)	ns	all ns[1], ns[2], ns[3]
Therapy - Sulfasalazin (%)	44.8	14.3	53.3	ns	p[1]=0.013, ns[2], ns[3]

Therapy - Biologicals (%) ***	6.9%	14.3	0	ns	ns[1], ns[2], ns[3]
Smokers ever/current (%)	37.9 / 37.9	50.0 / 50.0	26.6 / 26.6	ns	all ns[1], ns[2], ns[3]
BASDAI visit 1 / visit 2	2.18±0.36 / 2.46±0.41	1.79±0.37 / 1.91±0.50	2.55±0.61 / 2.98±0.63	ns	all ns[1], ns[2], ns[3]
BASFI visit 1 / visit 2	1.56±0.32 / 1.39±0.31	1.25±0.41 / 1.20±0.37	1.85±0.48 / 1.57±0.49	ns	all ns[1], ns[2], ns[3]
ASDAS-CRP visit 1 / visit 2	1.87±0.23 / 1.82±0.24	1.89±0.28 / 1.76±0.30	1.84±0.36 / 1.87±0.37	ns	all ns[1], ns[2], ns[3]
CRP visit 1/ visit 2 (mg/l)	7.72±1.47 /7.39±2.56	8.66±2.00 / 5.90±1.62	6.84±2.17 / 8.78±4.14	ns	all ns[1], ns[2], ns[3]

Abbreviations: axSpA- axial spondyloarthritis, AS- ankylosing spondylitis, nr-axSpA - nonradiographic axial spondyloarthritis, BMI - body mass index, NSA - non-steroidal antirheumatic drugs, BASDAI - Bath AS activity index, BASFI - Bath AS functional index, CRP - C reactive protein, ASDAS-CRP - AS disease activity score, TNFi - tumor necrosis factor alpha inhibitors

*All parameters were compared between all axSpA (1), AS (2) and nr-axSpA (3) in exercise group and group without intervention. Data for Exercise group see Table 1 and 2. All data are at baseline, only exacerbation and visit 2 after 6 months.

**Exacerbations of uveitis/arthritis occurring during 6 months of follow up

*** Only TNFi was used in long-term therapy for high disease activity.

Statistical analysis: The intergroup P value was calculated using either Mann-Whitney test or chi-squared/Fisher´s exact test. Data are characterised as mean ± standard deviation.
