# Supplementary material for: Clinical improvement and reduction in serum calprotectin levels after an intensive exercise programme for patients with ankylosing spondylitis and non-radiographic axial spondyloarthritis
Source: Arthritis Res Ther. 2016 Nov 25;18:275. doi: 10.1186/s13075-016-1180-1 (PMC5124318; doi:10.1186/s13075-016-1180-1)
Supplement: Additional file 2: — The dependence of the change in calprotectin levels on the changes in clinical and laboratory parameters. This table presents the regression model of the dependent variable ∆calprotectin and the independent variables of clinical and laboratory parameters. Only ∆CRP significantly influenced ∆calprotectin. (PDF 198 kb) [file 13075_2016_1180_MOESM2_ESM.pdf]

Additional File 2

The dependence of the change of caprotectin levels on the changes of clinical and laboratory parameters

	Regression coefficient	SE	Beta	t	*p*
(Constant)	-1146.372	1224.032		-0.937	0.353
**ΔCRP**	67.998	21.898	0.437	3.105	0.003
**ΔASDAS**	178.950	309.360	0.104	0.578	ns
**ΔBASDAI**	-79.945	138.255	-0.099	-0.578	ns
**ΔBASFI**	127.673	186.582	0.101	0.684	ns
**BMI baseline**	-4.341	39.475	-0.013	-0.110	ns
**axSpA with exercise/without exercise**	449.824	289.927	0.192	1.552	ns
**nr-axSpA/AS**	176.002	279.870	0.075	0.629	ns

Abbreviations: axSpA - axial spondyloarthritis, nr-axSpA - non radiographic axial spondyloarthritis, AS - ankylosing spondylitis, ASDAS-CRP - AS disease activity score, CRP - C-reactive protein, BASDAI- Bath AS disease activity index, Δ changes of the scores/biomarker values, SE -standard error, Beta - value of standardised coefficients, t- regression coefficients/SE ratio, ns - not significant, p - p value (statistical significance)

Statistical analysis: Regression model of dependent variable Δ calprotectin to independent variables Δ CRP, ΔASDAS-CRP, ΔBASDAI or ΔBASFI and BMI, axSpA without and with exercise intervention and nr-axSpA and AS. Explanatory value is r^2=30%. Significance was determined if $p<0.05$
